# Supplementary material for: Epidemiological and economic impact of COVID-19 in the US
Source: Sci Rep. 2021 Oct 14;11:20451. doi: 10.1038/s41598-021-99712-z (PMC8517017; doi:10.1038/s41598-021-99712-z)
Supplement: Supplementary file 1 — Supplementary Information. [file 41598_2021_99712_MOESM1_ESM.pdf]

Epidemiological and Economic Impact of COVID-19 in the US: Supplemental Information

Jiangzhuo Chen[1], Anil Vullikanti[1,2], Joost Santos[3], Srinivasan Venkatramanan[1], Stefan Hoops[1], Henning Mortveit[1,4], Bryan Lewis[1], Wen You[5], Stephen Eubank[1,5], Madhav Marathe[1,2], Chris Barrett[1,2], and Achla Marathe[1,5,*]

[1]Network Systems Science and Advanced Computing Division, Biocomplexity Institute, University of Virginia, Charlottesville, VA 22904, USA
[2]Department of Computer Science, University of Virginia
[3]Department of Engineering Management and Systems Engineering, George Washington University, Washington, DC 20052, USA
[4]Department of Engineering Systems and Environment, University of Virginia
[5]Department of Public Health Sciences, University of Virginia
[*]achla@virginia.edu

ABSTRACT

There is no abstract for this supplementary file.

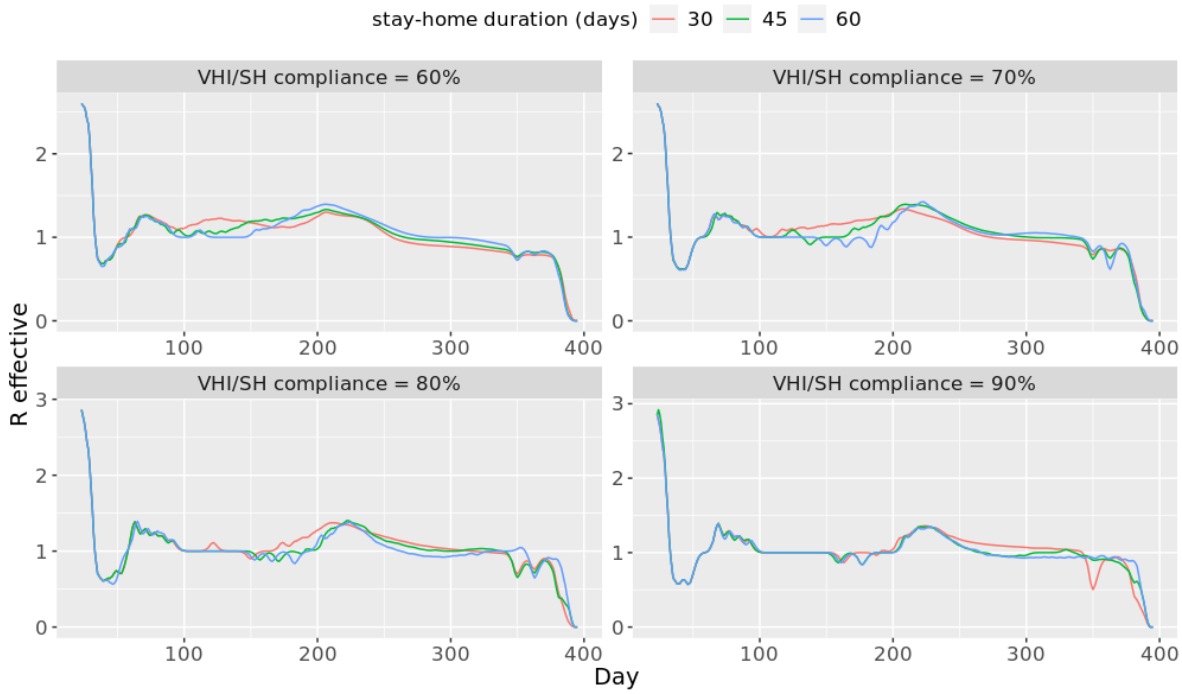

Figure 1A. $R_{\text{effective}}$, the effective reproduction number, under each scenario. We estimate $R_{\text{effective}}$ from the daily incidence time series generated by simulations, using an R package called `EpiEstim`.

The Disease Model Parameters

The CDC disease model in Figure 2 shows the state transitions which include transmissions and progressions. The former occurs when an individual in *Susceptible* state comes in contact with an individual in one of *Presymptomatic*, *Symptomatic*, or *Asymptomatic* states. The latter occurs when an individual has been in that state for a certain amount of time (called dwell time); the transitions are probabilistic. The dwell time distributions and the transition probability distributions are age dependent and are specified in the table.

Progression	Attribute	Age group				
		0-4	5-17	18-49	50-64	65+
Exposed - Asympt	prob	0.35				
Exposed - Asympt	dt-mean	5				
Exposed - Asympt	dt-std dev	1				
Asympt - Recovered	prob	1				
Asympt - Recovered	dt-mean	5				
Asympt - Recovered	dt-std dev	1				
Exposed - Presympt	prob	0.65				
Exposed - Presympt	dt-fixed	1				
Presympt - Sympt	prob	0.65				
Presympt - Sympt	dt-fixed	1				
Sympt - Attd	prob	0.9594	0.9894	0.9594	0.912	0.788
Sympt - Attd	dt-discrete	1:0.175, 2:0.175, 3:0.1, 4:0.1, 5:0.1, 6:0.1, 7:0.1, 8:0.05, 9:0.05, 10:0.05				
Attd - Recovered	prob	1				
Attd - Recovered	dt-mean	5				
Attd - Recovered	dt-std dev	1				
Sympt - Attd(D)	prob	0.0006	0.0006	0.0006	0.003	0.017
Sympt - Attd(D)	dt-fixed	2				
Attd(D) - Hosp(D)	prob	0.95				
Attd(D) - Hosp(D)	dt-fixed	2				
Hosp(D) - Vent(D)	prob	0.06	0.06	0.06	0.15	0.225
Hosp(D) - Vent(D)	dt-fixed	2				
Vent(D) - Death	prob	1				
Vent(D) - Death	dt-fixed	4				
Hosp(D) - Death	prob	0.94	0.94	0.94	0.85	0.775
Hosp(D) - Death	dt-fixed	6				
Attd(D) - Death	prob	0.05				
Attd(D) - Death	dt-fixed	8				
Sympt - Attd(H)	prob	0.04	0.01	0.04	0.085	0.195
Sympt - Attd(H)	dt-fixed	1				
Attd(H) - Hosp	prob	1				
Attd(H) - Hosp	dt-mean	5	5	5	5.3	4.2
Attd(H) - Hosp	dt-std dev	4.6	4.6	4.6	5.2	5.2
Hosp - Recovered	prob,	0.2				
Hosp - Recovered	dt-mean	3.1	3.1	3.1	7.8	6.5
Hosp - Recovered	dt-std dev	3.7	3.7	3.7	6.3	4.9
Hosp - Vent	prob	0.06	0.06	0.06	0.15	0.225
Hosp - Vent	dt-mean	1				
Hosp - Vent	dt-std dev	0.2				
Vent - Recovered	prob	1				
Vent - Recovered	dt-mean	2.1	2.1	2.1	6.8	5.5
Vent - Recovered	dt-std dev	3.7	3.7	3.7	6.3	4.9

Table 1A. Age dependent dwell-time distributions and transition probability distributions. Here “dt” refers to duration, e.g. dt-mean is mean duration and dt-discrete is discrete distribution duration. “Attd” is medically attended; “Hosp” is hospitalized; “Vent” is ventilated; “Sympt” is symptomatic and “Asympt” is asymptomatic.

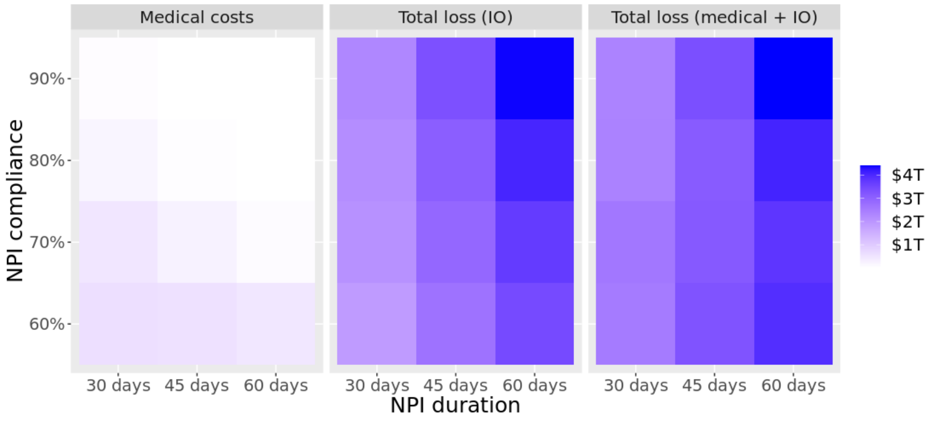

Figure 2A. A heatmap of costs under different NPI durations and compliance rates. The left panel shows the medical costs, the middle panel shows the input-output loss due to labor shock and interdependencies between industries, and the right panel shows the total loss.

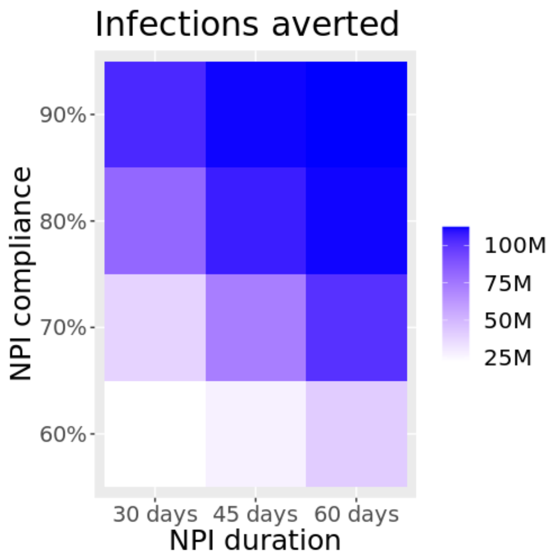

Figure 3A. Number of infections averted under each scenario.

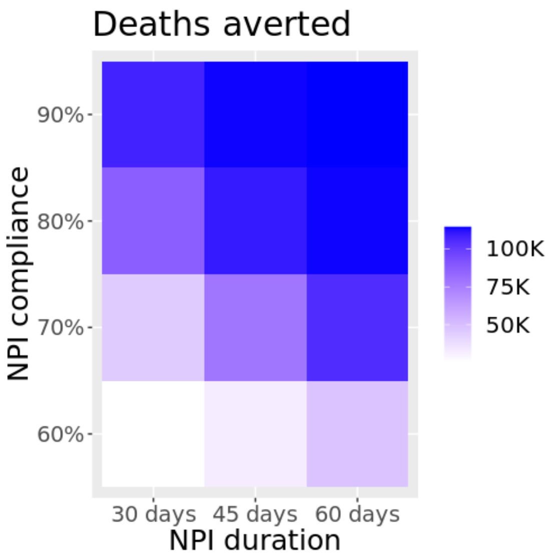

Figure 4A. Number of deaths averted under each scenario.
